# Deep parameter-free attention hashing for image retrieval

**DOI:** 10.1038/s41598-022-11217-5

**Published:** 2022-04-30

**Authors:** Wenjing Yang, Liejun Wang, Shuli Cheng

**Affiliations:** 1 College of Software, Xinjiang University, Urumqi, 830046 China; 2 College of Information Science and Engineering, Xinjiang University, Urumqi, 830046 China

**Keywords:** Electrical and electronic engineering, Computer science, Information technology, Software

## Abstract

Deep hashing method is widely applied in the field of image retrieval because of its advantages of low storage consumption and fast retrieval speed. There is a defect of insufficiency feature extraction when existing deep hashing method uses the convolutional neural network (CNN) to extract images semantic features. Some studies propose to add channel-based or spatial-based attention modules. However, embedding these modules into the network can increase the complexity of model and lead to over fitting in the training process. In this study, a novel deep parameter-free attention hashing (DPFAH) is proposed to solve these problems, that designs a parameter-free attention (PFA) module in ResNet18 network. PFA is a lightweight module that defines an energy function to measure the importance of each neuron and infers 3-D attention weights for feature map in a layer. A fast closed-form solution for this energy function proves that the PFA module does not add any parameters to the network. Otherwise, this paper designs a novel hashing framework that includes the hash codes learning branch and the classification branch to explore more label information. The like-binary codes are constrained by a regulation term to reduce the quantization error in the continuous relaxation. Experiments on CIFAR-10, NUS-WIDE and Imagenet-100 show that DPFAH method achieves better performance.

## Introduction

Recently, a great number of media data have been extensively used in various industries such as computer vision and network security^1,2^. Image retrieval in computer vision is the focus of current research. It is an urgent problem that quickly retrieve the similar image from a large data set. Due to the advantages of fast query speed and low storage cost, deep hashing method^3–7^ is widely applied in the field of image retrieval. The purpose of deep hashing is to convert high-dimensional images to low-dimensional binary codes by using a hash function, thereby preserving similar information of original images.

In the early image retrieval methods, text-based image retrieval (TBIR)^8,9^ follows the traditional text annotation technology to implement retrieval by text matching. In content-based image retrieval (CBIR)^10,11^, with the help of the computer to explore image content features and take it as clues to detect other images with similar features from image database. However, TBIR and CBIR need a great quantity manual operation and computational resources. On the contrary, deep hashing methods^12–14^ have obvious advantages by utilizing CNN as a features extractor. Existing deep hashing are divided into data-independent and data-dependent. In the data-independent hashing^15^, the hash codes are obtained by randomly mapping matrix and the accuracy of hash functions cannot be guaranteed. Data-dependent hashing^16,17^ explore multiple aspects of images such as shape, texture and colors to generate hash codes with discrimination ability. This study uses the data-dependent methods to learn high-quality hash codes.

Most current hashing methods commonly use the shallow CNN to explore high-dimensional semantic features and map them to hash codes via a hash function. However, the feature learning part of these methods have defects that features extraction is insufficiency and imbalance. Meanwhile, the hashing learning part cannot make full use of label information and produce insurmountable quantization errors, which significantly affect the accuracy of hash codes. Therefore, some scholars suggest adding channel-wise and spatial-wise attention mechanism to backbone network^18,19^. Such attention modules usually cause two problems. First, the flexibility of learning attention weights is hampered because they can only extract images features along channels or spatial dimensions. Second, their structures are composed of complicated factors, it will increase the complexity of the training model and cause over fitting.

**Figure 1:**
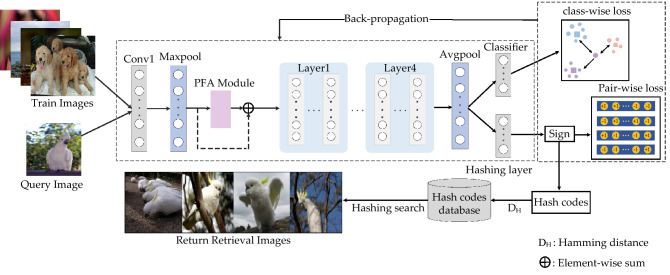
The overall framework of deep parameter-free attention hashing module, which is composed of three parts: (1) pairs of images are fed into Convolution and Maxpool layer to obtain feature map; (2) features map is fed into the PFA module, and the result obtained perform the element-wise sum operation with feature map; (3) a hashing layer is designed to generate hash codes, and three loss functions are used to optimize the network. (Created by ‘Microsoft Office Visio 2013’ url: https://www.microsoft.com/zh-cn/microsoft-365/previous-versions/microsoft-visio-2013).

To optimize the above problems, this paper is encouraged by 3-D attention module^20^ and semantic hierarchy preserving deep hashing^21^. This paper designs a parameter-free attention (PFA) module which defines an energy function that consider the weights of both channel and spatial dimensions. This module makes the network learn more differentiated neurons without adding parameters, and the high-level semantic features of the images can be fully explored through refine those neurons. Specifically, ResNet18 is chosen as backbone network. As shown in Fig. 1, the whole process is mainly divided into four steps. First, the pairs of images are fed into the Convolution layer and the Maximum pooling layer to generate feature map. Second, the feature map is processed by PFA module, which considers both the 1-D channel-wise weights and the 2-D spatial-wise weights and directly generated 3-D weights. Third, this paper performs the operation of element-wise sum on PFA output and feature map and input the result to the backbone network to extract image features. Finally, in order to make efficient use of semantic label information, two branches containing classifier layer and hashing layer is designed. Combining the pair-wise loss and quantization loss generated by the hashing layer and class-wise loss generated classifier to obtain hash codes with discriminative ability.

In short, the contributions are as follows:

- 1. DPFAH is an end-to-end learning framework which perform simultaneous feature representation and binary codes learning. A lightweight module is introduced to extract rich semantic features and avoid over fitting in the training process.
- 2. The PFA module is embedded in ResNet18 network to improve the feature representation. It explores an energy mathematical formula to calculate the 3-D weight and derives a closed-form solution that speedup the weight calculation. No parameters are added to the network during the whole process.
- 3. A novel deep hashing framework is designed by DPFAH, which includes hashing learning and classification. This method can use the label information to eliminate discrepancy and generate more accurate hash codes. Experimental results on three datasets have verified DPFAH.

The remaining content of this paper is as follows. “Related work” is related work. “Deep Parameter-free attention hashing” describes the details of DPFAH. “Experiments” is the results of experiments and analysis. “Conclusions” summarizes the work of this study.

## Related work

Deep learning is applied in many fields for its advantages of a solid learning ability and good portability. Network security fields use neural networks to detect malware^22,23^ and programs^24^, The field of artificial intelligence can be conducive to the intelligent estimation of traffic time by deep learning methods^25^. This paper focuses on the research of hashing algorithm based on deep learning. Deep hashing is widely applied in image retrieval system due to its own advantages. For example, the function of searching images by image is realized through deep hashing in many shopping software. Therefore, how to obtain hash code with strong accuracy for each image has become a research hotspot. In this section, the existing several unsupervised hashing approaches and supervised hashing approaches are introduced.

### Unsupervised hashing

Unsupervised hashing^26–30^ only utilizes the unlabeled data points to learn hash function that map high dimensional feature to compact hash codes. The similarity matrix is usually constructed in the process of feature learning. Many scholars have carried a lot of study on the perspective of constructing similarity matrix. Specifically, Sheng et al. proposed^28^ the descriptors of data are represented by the output of full-connected layer and used to design the similarity matrix. The network is optimized by calculating the loss between the similarity matrix and pairwise hash codes. By observing the law of features distribution, Yang et al. proposed^29^ the cosine distance of pairs data can be evaluated by Gaussian distributions. They set a distance threshold in the steps of constructing the similarity matrix, the data points are defined as similar if the cosine distance of data points smaller than threshold, vice versa. On this basis, Jiang et al. proposed^30^ the cosine distance was used directly to guide the construction of similarity matrix, and encouraged by^31^ , they chose the gradient attention to optimize the network. Although unsupervised hashing retrieval faces great challenges due to without labels information, these methods contribute to the development of image retrieval.

### Supervised hashing

Compared to unsupervised hashing, supervised hashing methods try to explore data labels as supervised information to calculate similarity matrix. Early on, Xia et al. proposed^32^ to learn semantic features and hash codes separately, and there is no feedback between them. Recent supervised hashing usually designs an end-to-end learning framework to learn features and hash codes simultaneously such as^31–34^. On this basis, Cao et al.^18^ selected a $tanh$ activation function that make the network output is continuous hash codes. To avoid the discrete limit imposed on like-binary codes, Su et al. proposed^35^ the greedy rules by updating the parameters toward the possible optimum discrete solution. In order to solve the problem of imbalanced distribution of data labels, Jiang et al.^36^ introduced a soft concept that quantified pairwise similarity as a percentage by using labels information. Meanwhile, Cao et al.^37^ proposed to weight the similarity matrix of training pairs and the Cauchy distribution is utilized instead of $sigmoid$ function to calculate the loss. These methods are improvements in the loss function, but they ignore the problem of insufficient image features extraction. Hence, Li et al.^19^ embedded channel attention and spatial attention into CNN to obtain sufficient semantic features. Yang et al.^34^ improved the feature map in the dual attention module and combined it with the backbone network. However, these modules can aggravate the complexity of the network model and affect the speed of training. Motivated by^38^, this paper introduces a lightweight attention module based on ResNet18 and design a new class-wise loss, which suitable for learning more accurate hash codes.

## Deep parameter-free attention hashing

In this section, the detail of DPFAH method is described, including research motivation, the definition of letters and formulas, the architecture of network, PFA module and the process of optimizing network.

### Research motivation

Recently, there are some defects in deep hashing method that need to be deal with: (1) shallow network cannot fully extract the semantic feature information of images, some channel-based or spatial-based attention modules can increase the complexity of model and lead to over fitting; (2) the process of relaxing hash codes can produce inevitable quantization error.

In order to solve the problem of insufficient feature extraction, some scholars consider adding attention mechanism modules to the network, which will increase the complexity of network computing and algorithm time complexity. Based on the above considerations, the goal of this paper is to design a lightweight module that can extract image features without adding any parameters to the network, and a regulation term constrained hash codes is proposed to reduce the quantization error.

### Problem formulation

In the similarity retrieval, given a dataset with $n$ images are represented as $X={\{{x}_{i}\}}_{i=1}^{n}$, where ${x}_{i}$ represents the $ith$ image. The label of $X$ is denoted as $Y={\{{y}_{i}\}}_{i=1}^{c}$, where ${y}_{i}$ is the labels of the $ith$ image and $c$ is the number of classes. Therefore, the similarity matrix $S=\{{s}_{ij}\}$ is defined as:

1 $${s}_{ij}=\left\{\begin{array}{l}1,\, \quad if \, {x}_{i}\,  and\,  {x}_{j}\,  belong\,  to \, the \, same\,  class \\ 0,  \quad\, otherwise\end{array}\right.$$

The target of deep hashing is to learn a hash function $F(\theta ;{x}_{i})$ that project ${x}_{i}$ to ${b}_{i}\in {\{-1,+1\}}^{l}$, where $\theta$ represents the parameters of CNN and $l$ is the length of hash codes. Therefore, each image ${x}_{i}$ is mapped to $l$-dimensional vector $U={\left\{{u}_{i}\right\}}_{i=1}^{n}$ passing through $F$ model, where ${u}_{i}$ is the $l$-dimensional vector of the $ith$ image. To reduce quantification loss, inspired by^34^, ${u}_{i}$ is processed by a piecewise function as follow:

2 $$f\left({u}_{i}\right)=\left\{\begin{array}{l}1,  \quad {u}_{i}>1\\ {u}_{i}, -1<{u}_{i}<1\\ -1,  \quad {u}_{i}<-1\end{array}\right.$$

Finally, ${b}_{i}=sign(f\left({u}_{i}\right))$ is used to map $l$-dimension ${u}_{i}$ to $l$-bit ${b}_{i}$, the $sign(.)$ is defined as follow:

3 $$sign\left(x\right)=\left\{\begin{array}{l}-1,  \quad x\ge 0\\ 1,  \quad otherwise\end{array}\right.$$

### Network architecture

Figure 1 shows the framework of DPFAH, which includes three main parts. DFPAH utilizes ResNet18 as backbone network, in order to fully improve the salient features representation ability and does not increase the computational complexity of the model. This paper has drawn a simple and parameter-free module into network, which can explore neurons in each channel or spatial location to learn more discriminative cues. In addition, the last layer of basic residual network is the classification layer that assigns data to the same class. On this basis, the hashing branch is designed parallel to the classification branch. The class-wise loss generated by the classification branch will positively affect the hashing branch when the parameters are updated by back propagation.

### PFA module

**Figure 2:**
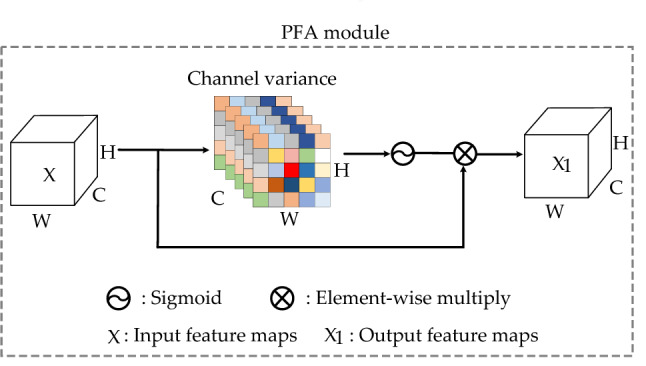
The detail of PFA module. The mean value of input feature maps $X$ and the channel variance are computed to judge the importance of each channel and spatial, so as to generate 3-D weights. Then 3-D weights are processed by the sigmoid activation function and multiplied by $X$ to obtain the output feature maps ${X}_{1}$. (Created by ‘Microsoft Office Visio 2013’ url: https://www.microsoft.com/zh-cn/microsoft-365/previous-versions/microsoft-visio-2013).

**Figure 3:**
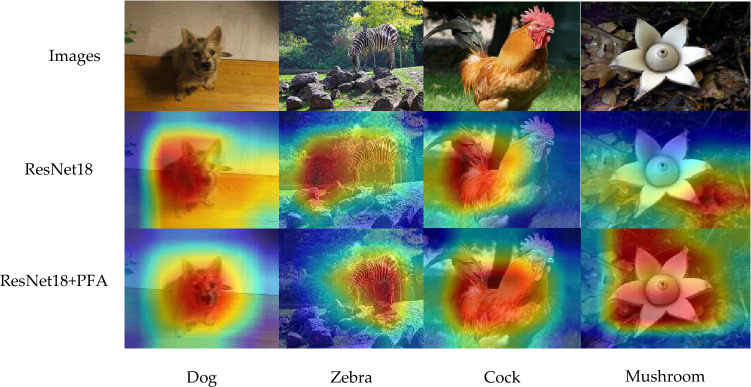
Visualization of feature activations. (Created by ‘Microsoft Office Visio 2013’ url: https://www.microsoft.com/zh-cn/microsoft-365/previous-versions/microsoft-visio-2013).

The existing attention modules consider the channel-wise attention or spatial-wise attention respectively. For channel-wise attention, the importance of each channel is firstly calculated from the perspective of channels, and then the channel with high importance is assigned greater 1-D weights. For spatial-wise attention, the importance of features at each location is calculated from a spatial point of view, and then the location with higher importance is assigned greater 2-D weights. These modules can increase the computational overhead when computing the 1-D or 2-D attention weights. Hence, this paper introduces a lightweight attention module (PFA) that can directly calculate 3-D weights. As shown in Fig. 2, first, the mean of $\overline{X }$ of feature maps $X$ is obtained and calculate the square of $X$ and $\overline{X }$ to get the variance. The variance is then divided by the feature map to obtain the variance of each channel, which is used to determine the variance of each channel and the importance of each spatial. Finally, the sigmoid function is used to restrict the result, and then multiplied with the original feature map $X$. In addition, the PFA module can focus on the primary areas close to the image label. As shown in Fig. 3, the second line represents the distribution of features extracted using the ResNet18 network, and the third line represents the PFA module is added to the network. The label of the first image is dog, only using the ResNet18 network to extract features will pay attention to many noises outside the label. After adding PFA, feature activations are mainly distributed around dog. It has the same effect on the second image. The third and fourth image focuses on more feature activations information about labels after adding PFA. Hence, the effectiveness of the PFA module is proved by the visualization of feature activation shown by Grad-CAM^39^.

Thanks to the PFA introduces an energy function that derives a closed-form solution, it does not add parameters to the network. Inspired by neuroscience theories^40^, the neurons with the most information are usually the ones that show different firing patterns from those around them, and then those important neurons should be given higher priority. The simplest means to discover these neurons is to compute the linear relationship between one target neuron and the others. Consequently, an energy function for each neuron is defined as follows:

4 $${e}_{t}\left({w}_{t},{b}_{t},y,{x}_{i}\right)=\left({y}_{t}-\widehat{t}\right)+\frac{1}{M-1}\sum_{i=1}^{M-1}{({y}_{0}-{\widehat{x}}_{i})}^{2}$$

where $t$ is target neuron and ${x}_{i}$ is surrounding neurons in each channel of feature map $X\in {\mathbb{R}}^{C\times H\times W}$, ${w}_{t}$ and ${b}_{t}$ are weight and bias, $i$ is the $ith$ spatial dimension, $M$ is the number of neurons on a channel and $M=H\times W$, $\widehat{t}={w}_{t}t+{b}_{t}$ and ${\widehat{x}}_{i}={w}_{t}{x}_{i}+{b}_{t}$ are linear transforms of $t$ and ${x}_{i}$. ${y}_{t}$ and ${y}_{0}$ is the output of target neuron and surrounding neurons respectively and ${y}_{t}\ne {y}_{0}$. The minimum value is gained by Eq. (4) when ${y}_{t}=\widehat{t}$ and ${y}_{0}={\widehat{x}}_{i}$. In a channel, the linear separability between target neuron and other neurons can be obtained by calculating the minimum value of Eq. (4). For simplicity, this paper adopts ${y}_{t}=1$ and ${y}_{0}=-1$, add a regularization term to optimize the function. The energy function is transformed as follows:

5 $${e}_{t}\left({w}_{t},{b}_{t},y,{x}_{i}\right)=\frac{1}{M-1}\sum_{i=1}^{M-1}{(-1-({w}_{t}{x}_{i}+{b}_{t}))}^{2}+{(1-({w}_{t}{x}_{i}+{b}_{t}))}^{2}+\lambda {w}_{t}^{2}$$

There are $M$ energy functions on each channel, which are quite complex in calculation by using iterative. Luckily, Eq. (5) has a fast closed-form solution with respect to ${w}_{t}$ and ${b}_{t}$ as follows:

6 $${w}_{t}=-\frac{2\left(t-{\mu }_{t}\right)}{{\left(t-{\mu }_{t}\right)}^{2}+2{\sigma }_{t}^{2}+2\lambda }$$

7 $${b}_{t}=-\frac{1}{2}\left(t+{\mu }_{t}\right){w}_{t}$$

where ${\mu }_{t}$=$\frac{1}{M-1}{\sum }_{i=1}^{M-1}{x}_{i}$ and ${\sigma }_{t}^{2}=\frac{1}{M-1}{\sum }_{i=1}^{M-1}{{(x}_{i}-{\mu }_{t})}^{2}$ represents the mean and variance of surrounding neurons, respectively. Thanks to the solutions of Eqs. (6) and (7) are calculated on a single channel. This supposes that all features in a single channel follows the same distribution. The mean and variances of all neurons can be computed according to this suppose. This method considerably reduces the calculation cost. Therefore, the minimal energy can be computed as follows:

8 $${e}_{t}^{*}=\frac{4({\widehat{\sigma }}^{2}+\lambda )}{{\left(t-\widehat{\mu }\right)}^{2}+2{\widehat{\sigma }}^{2}+2\lambda }$$

where $\widehat{\mu }=\frac{1}{M}{\sum }_{i=1}^{M-1}{x}_{i}$ and ${\widehat{\sigma }}^{2}=\frac{1}{M}{\sum }_{i=1}^{M-1}{{(x}_{i}-\widehat{\mu })}^{2}$. From Eq. $(8)$, it can be concluded that the greater the difference between the target neuron and the surrounding neurons, the lower the energy function and the more stable the model will be. Although ${e}_{t}^{*}$ can represent the importance of each neuron, this method needs to calculate a large number of covariance matrix. Hence, this paper utilizes a scaling operator instead of an addition for feature refinement as follows:

9 $$\tilde{X }=sigmoid\left(\frac{1}{E}\right) \odot X$$

where $E$ group all ${e}_{t}^{*}$ across channel and spatial dimensions. Adding a $sigmoid$ function to prevent the value of $E$ from being too large.

### Model formulation

Input a pair of images ${x}_{i}$ and ${x}_{j}$ into the network to generate hash codes ${b}_{i}$ and ${b}_{j}$. The Hamming distance between ${b}_{i}$ and ${b}_{j}$ is defined as ${D}_{H}=\frac{1}{2}(l-\langle {b}_{i},{b}_{j}\rangle )$, where $\langle {b}_{i},{b}_{j}\rangle$ is the inner product and $l$ is the length of hash codes. It can be seen that there are opposite changes between inner product and Hamming distance. The larger ${D}_{H}$, the smaller $\langle {b}_{i},{b}_{j}\rangle$, and vice versa. Hence, the inner product is used instead of hamming distance to judge the similarity of pairwise images.

Given the set $B=[{b}_{1},{b}_{2},\dots ,{b}_{n}]$ of hash codes. The Maximum Likelihood estimation of $B$ for dataset $X$ is defined as follows:

10 $$logP\left(S|B\right)=\prod_{{s}_{ij}\in S}logP({s}_{ij}|B)$$

where $P\left(S|B\right)$ represents the likelihood function. For each image pair, $P\left({s}_{ij}|{b}_{i},{b}_{j}\right)$ is the conditional probability of ${s}_{ij}$ under the given premise of ${b}_{i}$ and ${b}_{j}$, which is calculated as follows:

$$P\left({s}_{ij}|{b}_{i},{b}_{j}\right)=\left\{\begin{array}{l}\sigma \left(\langle {u}_{i},{u}_{j}\rangle \right), \quad  {s}_{ij}=1 \\ 1-\sigma \left(\langle {u}_{i},{u}_{j}\rangle \right), \quad  {s}_{ij}=0 \end{array}\right.$$

11 $$={\sigma \left(\langle {u}_{i},{u}_{j}\rangle \right)}^{{s}_{ij}}{(1-\sigma \left(\langle {u}_{i},{u}_{j}\rangle \right))}^{1-{s}_{ij}}$$

where $\sigma \left(\cdot \right)$ is $sigmoid$ function defined as $\sigma \left(x\right)=\frac{1}{1+{e}^{-x}}$ and ${b}_{i}=sign\left({u}_{i}\right)$. The reason why this paper uses ${u}_{i}$ instead of ${b}_{i}$ is that ${b}_{i}$ will cause a discrete optimization problem in Eq. (11). ${u}_{i}$ is the continuous like-binary codes output by the network, which can avoid this problem.

Learning hash codes by combing Eqs. (10) and (11) as follows:

$${L}_{1}=-\mathit{log}P\left(S|B\right)=-\sum_{{s}_{ij}\in S}logP\left({s}_{ij}|B\right)$$

12 $$=-\sum_{{s}_{ij}\in S}({s}_{ij}\langle {u}_{i},{u}_{j}\rangle -log(1+exp(\langle {u}_{i},{u}_{j}\rangle )))$$

Equation (12) is the negative log likelihood loss function that shows the inner product of similar images should be as large as possible, the inner product of dissimilar images should be as small as possible. In other words, the hash codes of similar images are similar, and vice versa. Consequently, the hash codes preserve the similarity relation of the images in the original space.

In addition, there is an inevitable quantization error when ${u}_{i}$ is quantized to ${b}_{i}$. To solve this problem, inspired by^9^, this paper has made the following improvements to ${u}_{i}$:

13 $${L}_{2}=\sum_{i=1}^{n}ReLU\left(-\delta -{u}_{i}\right)+ReLU\left({u}_{i}-\delta \right)$$

where $ReLU\left(x\right)=\mathrm{max}(0,x)$ is the Rectified Linear Unit. This paper follows the optimization policy proposed by^34^, which relax ${u}_{i}$ to $[-\delta ,\delta ]$ and $\delta$ is set to 1.1.

Finally, in the classification layer, the output nodes of the network are determined by $c$ that is the number of categories in the dataset. The loss between the output of the classification layer and the label ${y}_{i}$ is defined as:

14 $${L}_{3}=-\sum_{i=1}^{n}{y}_{i}\mathit{log}\left(\frac{1}{1+{e}^{{-o}_{i}}}\right)+\left(1-{y}_{i}\right)\mathit{log}\left(\frac{{e}^{-{o}_{i}}}{1+{e}^{-{o}_{i}}}\right)$$

$$=-\sum_{i=1}^{n}\{{o}_{i}-{y}_{i}{o}_{i}+\mathit{log}\left(1+{e}^{{-o}_{i}}\right)\}$$

Additionally, ${o}_{i}$ is the real-valued classification layer outputs of the $ith$ image. By calculating Eq. (14), the generated hash codes by hashing layer saves classification information at the same time.

Overall, combing Eqs. (12), (13) and (14), the total loss of the framework model is expressed as:

15 $${L}_{all}={L}_{1}+\eta {L}_{2}+\zeta {L}_{3}$$

### Learning

The network parameters are optimized by calculating the gradient of the loss function and completing the back propagation. To learning a hash function for mapping images to hash codes, $\theta$ stands for the parameters of all feature layers, $\varphi ({x}_{i};\theta )$ denotes the output of network, ${W}^{T}\epsilon {\mathbb{R}}^{512\times l}$ is the transpose of the weight matrix and $v\in {\mathbb{R}}^{l\times 1}$ represents bias vector. A fully connected layer is employed to connect feature representation and hashing learning. It is set:

16 $${u}_{i}={W}^{T}\varphi \left({x}_{i};\theta \right)+v$$

In the DPFAH model, the parameters to be optimized are $\theta$, $W$, $v$ and ${b}_{i}$. The control variables method is adopted to optimize the parameters. Among them, ${b}_{i}$ can be directly optimized:

17 $${b}_{i}=sign\left({u}_{i}\right)$$

Before optimizing the parameters $\theta$, $W$ and $v$, this paper calculates the derivative of ${L}_{all}$ with respect to ${u}_{i}$ and ${o}_{i}$ by Eq. (15) as:

18 $$\frac{\partial {L}_{all}}{\partial {u}_{i}}=\frac{1}{2}\sum_{j:{s}_{ij}\in S}{(a}_{ij}-{s}_{ij}){u}_{j}+\frac{1}{2}\sum_{j:{s}_{ji}\in S}{(a}_{ji}-{s}_{ji}){u}_{j}+\eta \frac{\partial {L}_{2}}{\partial {u}_{i}}$$

where,

19 $$\frac{\partial {L}_{2}}{\partial {u}_{i}}=\left\{\begin{array}{l}n, \quad {u}_{i}\ge \delta \\ 0,\quad -\delta <{u}_{i}<\delta \\ -n, \quad {u}_{i}\le -\delta \end{array}\right.$$

20 $$\frac{\partial {L}_{all}}{\partial {o}_{i}}=\zeta (1-{y}_{i}-\frac{{e}^{{-o}_{i}}}{1+{e}^{{-o}_{i}}})$$

Then, this paper updates the parameters $W$ and $v$ by using back propagation:

21 $$\frac{\partial {L}_{all}}{\partial W}=\varphi \left({x}_{i};\theta \right){(\frac{\partial {L}_{all}}{\partial {u}_{i}})}^{T}$$

22 $$\frac{\partial {L}_{all}}{\partial v}=\frac{\partial {L}_{all}}{\partial {u}_{i}}$$

When optimizing network parameters, ${l}_{3}$ has a certain impact on parameter during back propagation, according to Eqs. (18) and (20), the gradient of $\theta$ is calculated as:

23 $$\frac{\partial {L}_{all}}{\partial \varphi \left({x}_{i};\theta \right)}=W\left(\frac{\partial {L}_{all}}{\partial {u}_{i}}+\frac{\partial {L}_{all}}{\partial {o}_{i}}\right)$$

The training process of the DPFAH model is exhibited in Algorithm 1.

**Figure:**
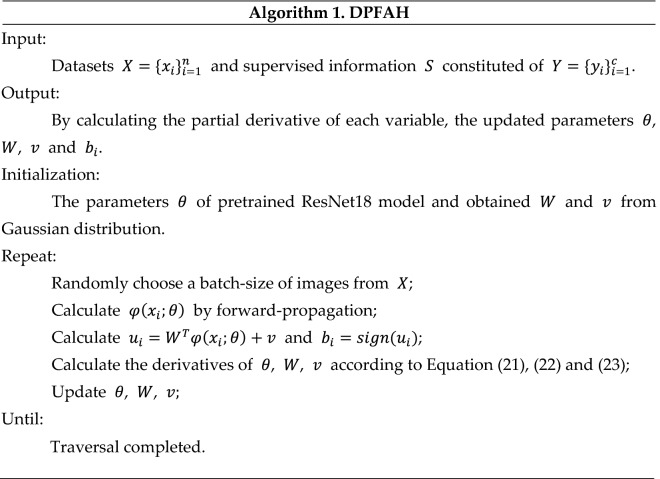

## Experiments

In this section, the DPFAH model is measured on three datasets. This paper compares the evaluation indexes of the DPFAH with the latest approaches.

### Datasets

(1) CIFAR-10 is a single-label public dataset, which include 60,000 images belonging to 10 classes, and each class have 6000 images. In this experiment, the training set is composed by selecting 500 images at random in each class, the testing set is formed by 100 images in each class. The remaining images are treated as the database. (2) NUS-WIDE is a multi-label public dataset including 269,648 images, this experiment selects 195,834 images belonging to 21 categories from them. Specifically, 100 images from each category form the testing set and the rest of images serve as the dataset. This experiments randomly select 500 images in each class as training set from the dataset. (3) Imagenet-100 is a single-label public dataset with 138,503 images and each image belongs to one of 100 classes. In experiment, the testing set is formed by 5000 randomly selected images, and the rest of the images serve as the database. At the same time, 130 images from each class of the dataset are chose as training set. In addition, the above three datasets are open-source datasets. All the procedures were performed in accordance with the relevant guidelines and regulations.

### Evaluation metrics and settings

There are four evaluation metrics in the experiment to measure the performance of DPFAH: mean average precision (mAP), precision-recall curves (PR), precision curves within Hamming distance 2 (P@H = 2) and precision curves of the first 1000 retrieval results (P@N). In addition, this paper selects mAP@ALL for CIFAR-10, mAP@5000 for NUS-WIDE and mAP@1000 for Imagenet-100. In order to prove the performance of DPFAH, the methods of DBDH^14^, DSDH^5^, DHN^4^, LCDSH^6^, HashNet^18^, IDHN^7^, DFH^13^ and DSH^3^ are selected for comparative experiment.

To make the experimental results objective and impartial, all comparative experiments are carried out on ResNet18 network and the Pytorch framework. Moreover, the parameter information of ResNet18 in each layer is shown in Table 1. Specifically, $p$ is the size of the convolution kernel, $s$ and $k$ represent the stride and padding, respectively, and $l$ is the length of hash codes.

**Table 1:** Configuration of ResNet18 network.

Layer	Configuretion
Conv1	$\{64\times 112\times 112, k=7\times 7, s=2\times 2, p=3\times 3, ReLU\}$
Maxpool	$\{64\times 54\times 54, k=3\times 3, s=2\times 2, p=1\times 1, ReLU\}$
Layer1	$\{64\times 56\times 56, k=3\times 3, s=1\times 1, p=1\times 1, ReLU\}\times 4$
Layer2	$\{128\times 28\times 28, k=3\times 3, s=2\times 2, p=1\times 1, ReLU\}\times 4$
Layer3	$\{256\times 14\times 14, k=3\times 3, s=2\times 2, p=1\times 1, ReLU\}\times 4$
Layer4	$\{512\times 7\times 7, k=3\times 3, s=2\times 2, p=1\times 1, ReLU\}\times 4$
Avgpool	$512\times 1\times 1$
Hashing Layer	$l$, the length of hash codes

In experiment, all comparative approaches use the same training set and testing set. The optimizer uses the root mean square prop (RMSProp), the mini batch size is set as 128, the learning rate is set as $5\times {10}^{-5}$ and the weight decay is set as $1\times {10}^{-5}$. The environment configuration is shown in Table 2.

**Table 2:** Environment configuration.

Item	Configuration
OS	Ubuntu 16.04($\times$ 64)
GPU	Tesla V100

### Hyperparameter analysis

In Eq. (15), this paper uses two hyperparameters $\eta$ and $\zeta$ to weigh the impact of classification loss and quantization loss on network optimization. The values of $\eta$ and $\zeta$ are determined by experimental results, as shown in Tables 3 and 4. This paper selects single-label dataset CIFAR-10 and multi-label dataset NUS-WIDE for parameter adjustment. Experiment fixes $\eta =10$ when adjusting $\zeta$. Similarly, experiment fixes $\zeta =0.1$ when adjusting $\eta$.

**Table 3:** mAP for of different $\zeta$.

$\zeta$	CIFAR-10 (mAP@ALL)				NUS-WIDE (mAP@5000)			
	16 bit	32 bit	48 bit	64 bit	16 bit	32 bit	48 bit	64 bit
0.05	0.7929	0.8161	0.8445	0.8264	0.8246	0.8442	0.8506	0.8538
0.1	0.8382	0.8445	0.8522	0.8549	0.8288	0.8490	0.8541	0.8580
0.5	0.8128	0.8123	0.8293	0.8444	0.8104	0.8407	0.8516	0.8534
1.0	0.8077	0.8285	0.8208	0.8363	0.8013	0.8342	0.8406	0.8436

**Table 4:** mAP for of different $\eta$.

$\eta$	CIFAR-10 (mAP@ALL)				NUS-WIDE (mAP@5000)			
	16 bit	32 bit	48 bit	64 bit	16 bit	32 bit	48 bit	64 bit
1	0.8134	0.8331	0.8345	0.8338	0.8238	0.8456	0.8517	0.8550
5	0.8237	0.8168	0.8239	0.8395	0.8232	0.8475	0.8547	0.8593
10	0.8382	0.8445	0.8522	0.8549	0.8288	0.8490	0.8541	0.8580
15	0.8153	0.8173	0.8377	0.8391	0.8160	0.8430	0.8512	0.8541

As shown in Table 3, the value of mAP is the largest on the two datasets when $\zeta =0.1$. The mAP decreases significantly when $\zeta =0.05$ on CIFAR-10, and mAP on NUS-WIDE is also decreasing slightly. Compared with $\zeta =0.1$, the mAP value of $\zeta =0.5$ decreased by 2.3% and 0.8% on average respectively on CIFAR-10 and NUS-WIDE. When ζ = 1, map values decreased by an average of 2.4% and 1.8% on two datasets. Therefore, it is concluded that when the hyperparameter $\zeta$ of class-wise loss is 0.1, the experimental result is better.

Figure 3 shows mAP on different $\zeta$ more intuitively, the mAP curves reach the peak when the value of a is 0.1. As the value of $\zeta$ becomes larger or smaller, the value of mAP will decrease slightly. Therefore, this paper chooses $\zeta =0.1$ to achieve the optimal experimental effect.

**Figure 4:**
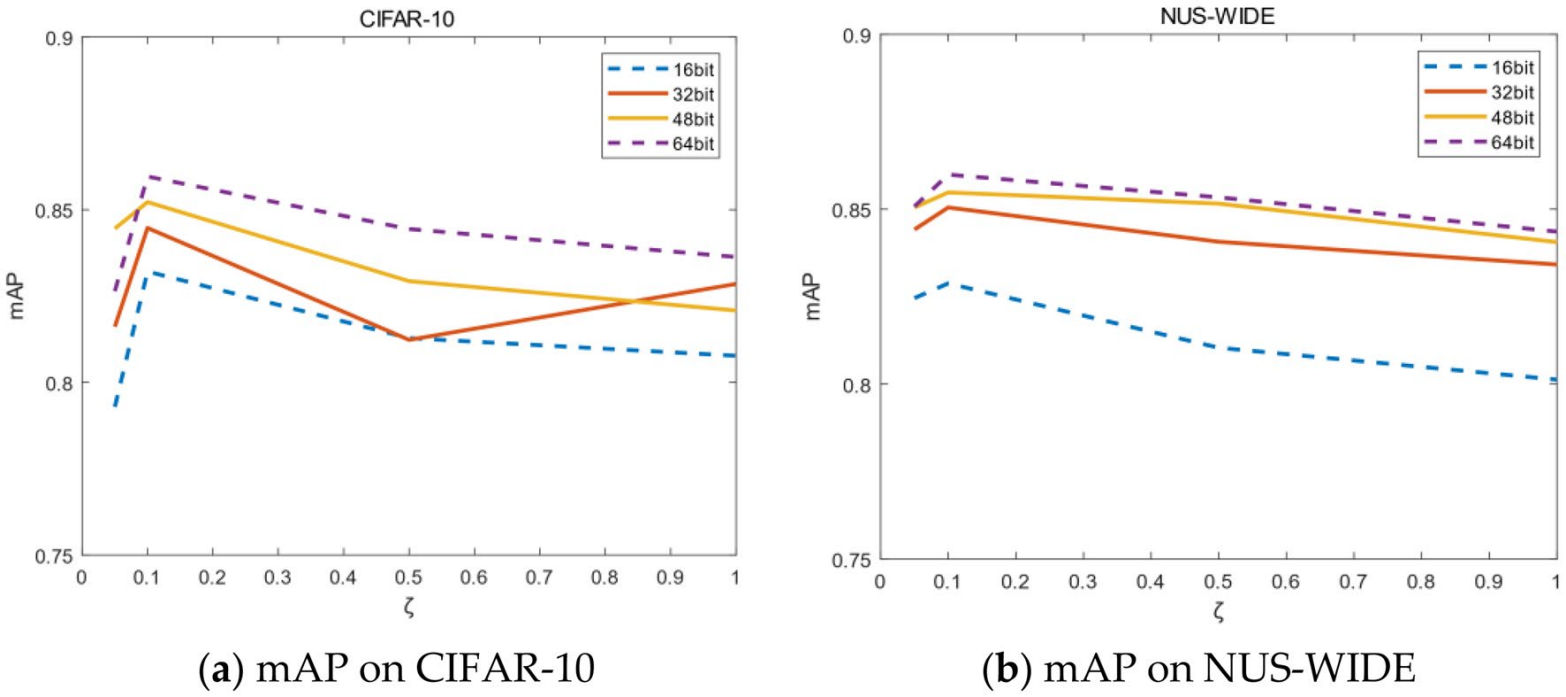
(**a,b**) Represent mAP on different $\zeta$. (Created by “matlab R2019a” url: https://ww2.mathworks.cn/products/matlab.html).

As shown in Table 4, When $\eta =10$, mAP reaches its maximum value. On CIFAR-10 and NUS-WIDE, compared with $\eta =10$, the mAP value of $\eta =1$ decreased by 1.9% and 0.3% on average, the mAP value of $\eta =5$ decreased by 2.1% and 0.1% on average, and the mAP value of $\eta =15$ decreased by 2.0% and 0.6% on average respectively. Therefore, when the hyperparameter $\eta$ of quantization loss is 10, good results can be obtained in the experiment.

**Figure 5:**
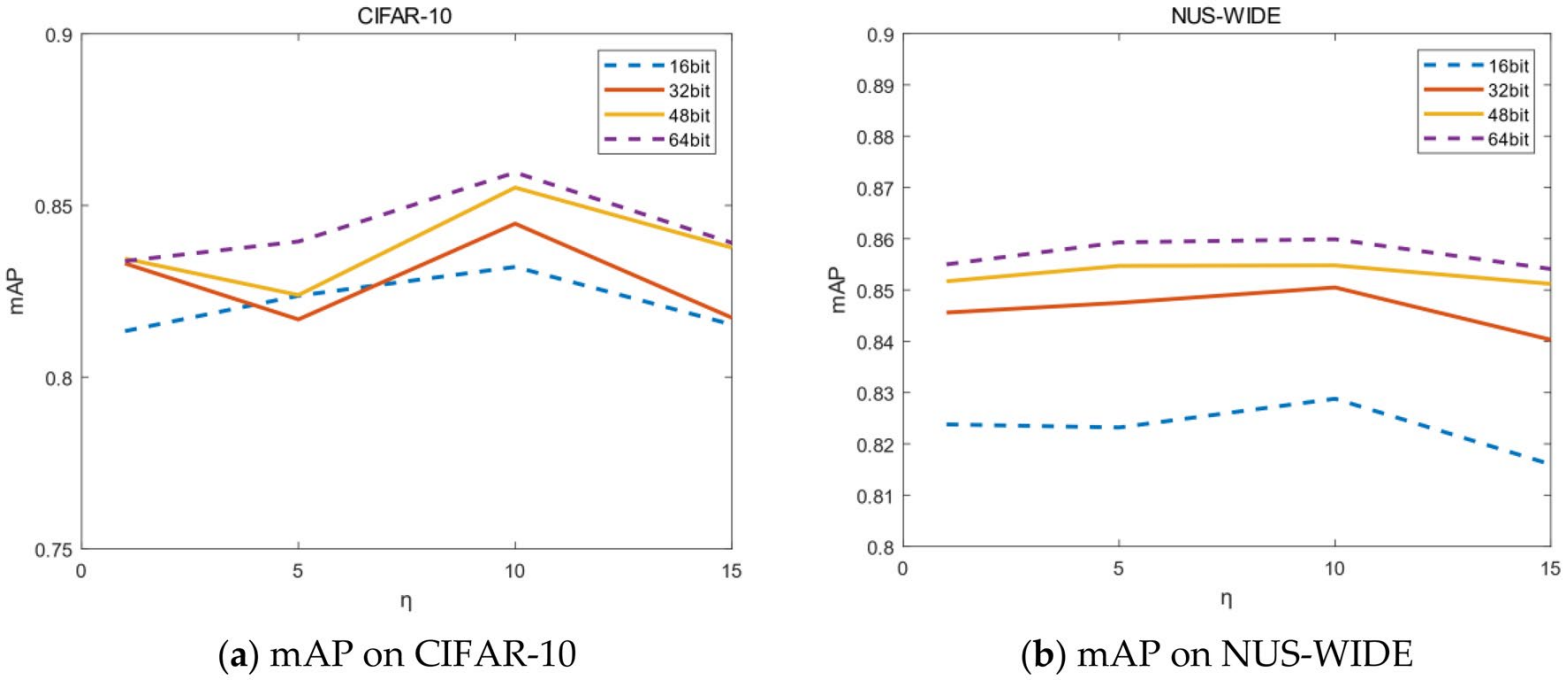
(**a,b**) Represent mAP on different $\zeta$. (Created by “matlab R2019a” url: https://ww2.mathworks.cn/products/matlab.html).

Similarly, as shown in Fig. 5, the value of mAP is higher than the others when $\eta =10$, and the mAP curves reach the peak on CIFAR-10. On NUS-WIDE, when $\eta$ takes 5 and 10, the mAP at 48 bit and 64 bit are close, but the mAP value of $\eta =10$ is significantly better than $\eta =5$ at 16 bit and 32 bit. Therefore, this paper also sets $\eta$ as 10 to achieve optimal experimental effect.

### Empirical analysis

In order to fully extract image features without increasing network complexity, this paper adds the PFA module to ResNet18 network, which can extract 3-D weights of features. Compared with the common attention mechanism module, the structure of PFA is simple and parameters-free. Meanwhile, to improve the discrimination and accuracy of hash codes, this paper designs classification branches in the network. Equation (13) is designed to reduce quantization errors. Equation (14) is the class-wise loss generated by the classification layer. Equations (13) and (14) are integrated to ${L}_{loss}$ in ablation experiments. As shown in Table 5, DBDH is selected as the baseline and the length of hash codes is 48 bit on CFIAR-10 dataset. DBDH indicates the baseline model utilizing AlexNet network. DFPAH-1 chooses ResNet18 as backbone instead of AlexNet. On this basis, DFPAH-2 shows that PFA Module has been added to the network. DFPAH-3 adds ${L}_{loss}$ to the network. The symbol √ indicates adding corresponding module.

**Table 5:** Ablation experiments.

Modules	DBDH	DFPAH-1	DFPAH-2	DFPAH-3
Alexnet	√			
ResNet18		√	√	√
PFA Module			√	√
${L}_{loss}$				√
mAP(48bit)	0.7839	0.8129	0.8424	0.8522

As shown in Table 5, PFA module is added on the basis of DFPAH-1, and the mAP value is increased by 2.95%, which proves that PFA module improves the accuracy of image retrieval. The mAP value of DFPAH-3 is up to 0.98% higher than DFPAH-2, showing the effectiveness of ${L}_{loss}$.

**Figure 6:**
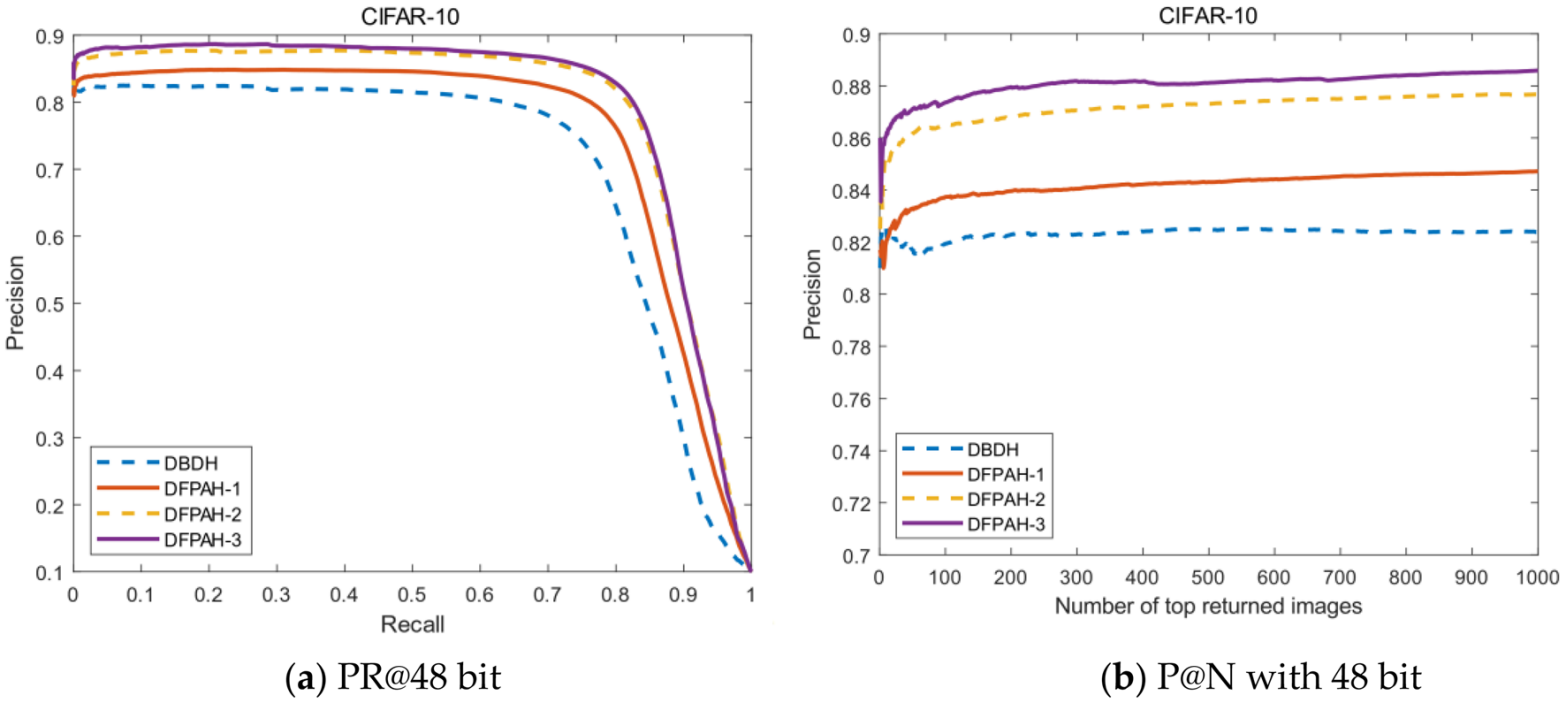
(**a,b**) Present the PR curves and P@N curves, respectively. (Created by “matlab R2019a” url: https://ww2.mathworks.cn/products/matlab.html).

Figure 6a intuitively shows the PR curves added with PFA module and ${L}_{loss}$, which is significantly higher than the baseline model. Figure 6b displays the precision of returning the first 1000 images, DFPAH-3 is obviously better than others. Hence, the above ablation experiments verify the effectiveness of PFA module and ${L}_{loss}$.

### Visualization of hash codes by t-SNE

**Figure 7:**
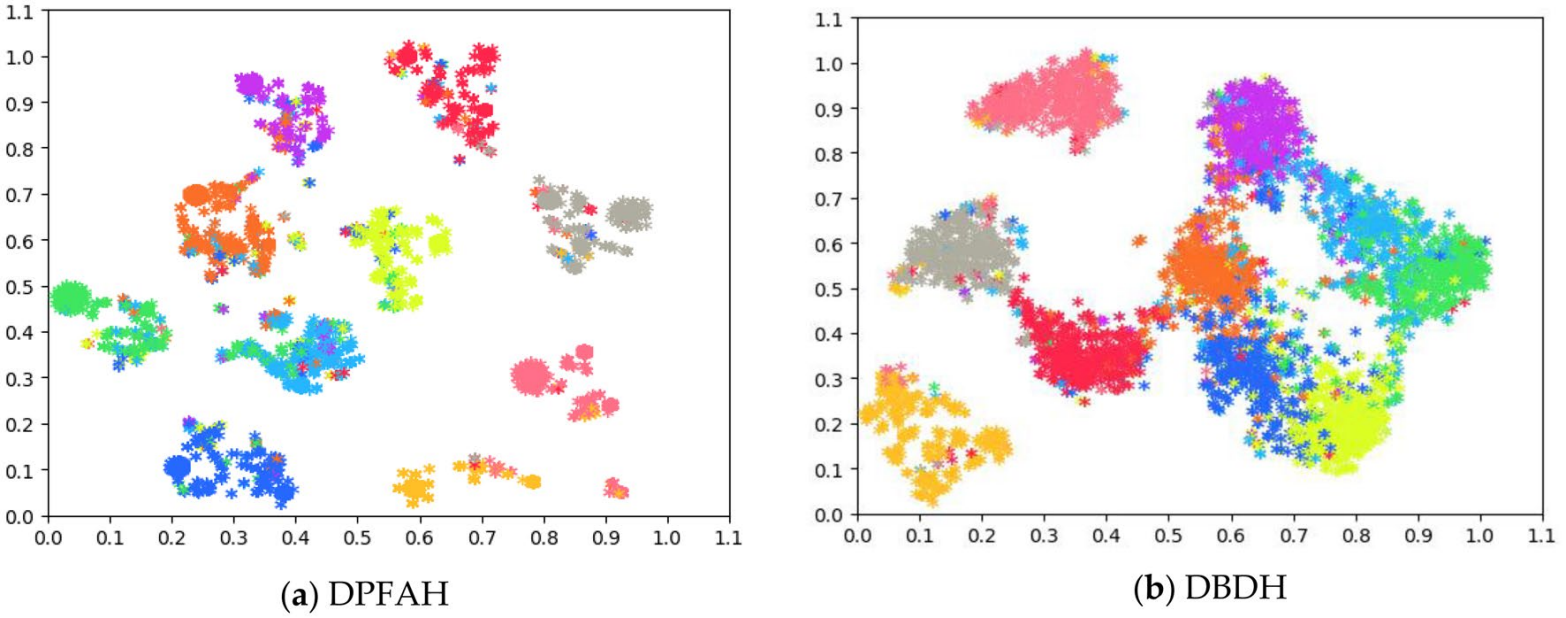
(**a,b**) Present the t-SNE visualization of hash codes on CIFAR-10. (Created by “python3.6” https://www.python.org/downloads/release/python-3614).

Figure 7 shows the t-SNE Visualization of the hash codes learned by DPFAH and the baseline DBDH on CIFAR-10 dataset. As shown in Fig. 7a, the hash codes generated by DPFAH show clear discriminative structures where the hash codes in different categories are well separated, while the hash codes generated by DBDH do not show such clear structures. This verifies that by introducing the PFA module and ${L}_{loss}$ for hashing, the hash codes generated through DPFAH are more discriminative than that generated by DBDH. Therefore, DPFAH method effectively increases the spacing between inter classes and reduces the gap intra classes, making the generated hash codes compact and effectively enhancing the representation ability.

### Results analysis

As shown in Table 6, it shows the mAP results of all comparative experiments on the CIFAR-10, NUS-WIDE and Imagenet-100. Experiments select the length of hash codes from 16 to 64 bit. The mAP of DPFAH have reached 83.82%, 84.45%, 85.22% and 85.49% on the CIFAR-10, which improved by an average of 3.57% compared to the baseline model. On the NUS-WIDE dataset, the mAP of DPFAH in different hash codes length achieves 82.98%, 84.90%, 85.41% and 85.80%. Compared with the classic methods DHN on the CIFAR-10, DPFAH have improved by 6.87%, 5.74%, 6.53% and 5.83% respectively. On the NUS-WIDE dataset, DPFAH achieves 1.90%, 4.21%, 6.87% and 6.70% growth compared with DHN on different bits. On the Imagenet-100 dataset, the effect of DPFAH is the most obvious in three datasets, compared with baseline model DBDH, DPFAH has achieves 30.62%, 37.94%, 15.86% and 14.74% on different bits. Hence, a large number of experiments show that the model trained by DPFAH has higher robustness.

**Table 6:** mAP for different bit on three datasets.

Method	CIFAR-10 (mAP@ALL)				NUS-WIDE (mAP@5000)				Imagenet-100 (mAP@1000)			
	16bit	32bit	48bit	64bit	16bit	32bit	48bit	64bit	16bit	32bit	48bit	64bit
DPFAH	0.8382	0.8445	0.8522	0.8549	0.8298	0.8490	0.8541	0.8580	0.6420	0.7009	0.7212	0.7795
DBDH	0.8021	0.8113	0.8129	0.8209	0.8084	0.8345	0.8393	0.8492	0.3358	0.3215	0.5626	0.6321
DSDH	0.7761	0.7881	0.8086	0.8183	0.8085	0.8373	0.8265	0.8441	0.1612	0.3011	0.3638	0.4268
DHN	0.7695	0.7871	0.7869	0.7966	0.8108	0.8069	0.7854	0.7910	0.4900	0.4808	0.4747	0.5664
LCDSH	0.7383	0.7661	0.8083	0.8202	0.8071	0.8304	0.8425	0.8436	0.2269	0.3177	0.4517	0.4671
Hashnet	0.6975	0.7892	0.7878	0.7949	0.7453	0.8004	0.8268	0.8297	0.3017	0.4690	0.5400	0.5719
IDHN	0.6641	0.7296	0.7762	0.7681	0.7820	0.7795	0.7601	0.7366	0.2721	0.3255	0.4477	0.5539
DFH	0.5947	0.6347	0.7298	0.7662	0.7893	0.8185	0.8350	0.8372	0.1727	0.3435	0.3445	0.3430
DSH	0.5095	0.4663	0.4702	0.4714	0.6680	0.7383	0.7563	0.7940	0.3109	0.3848	0.4294	0.4403

**Figure 8:**
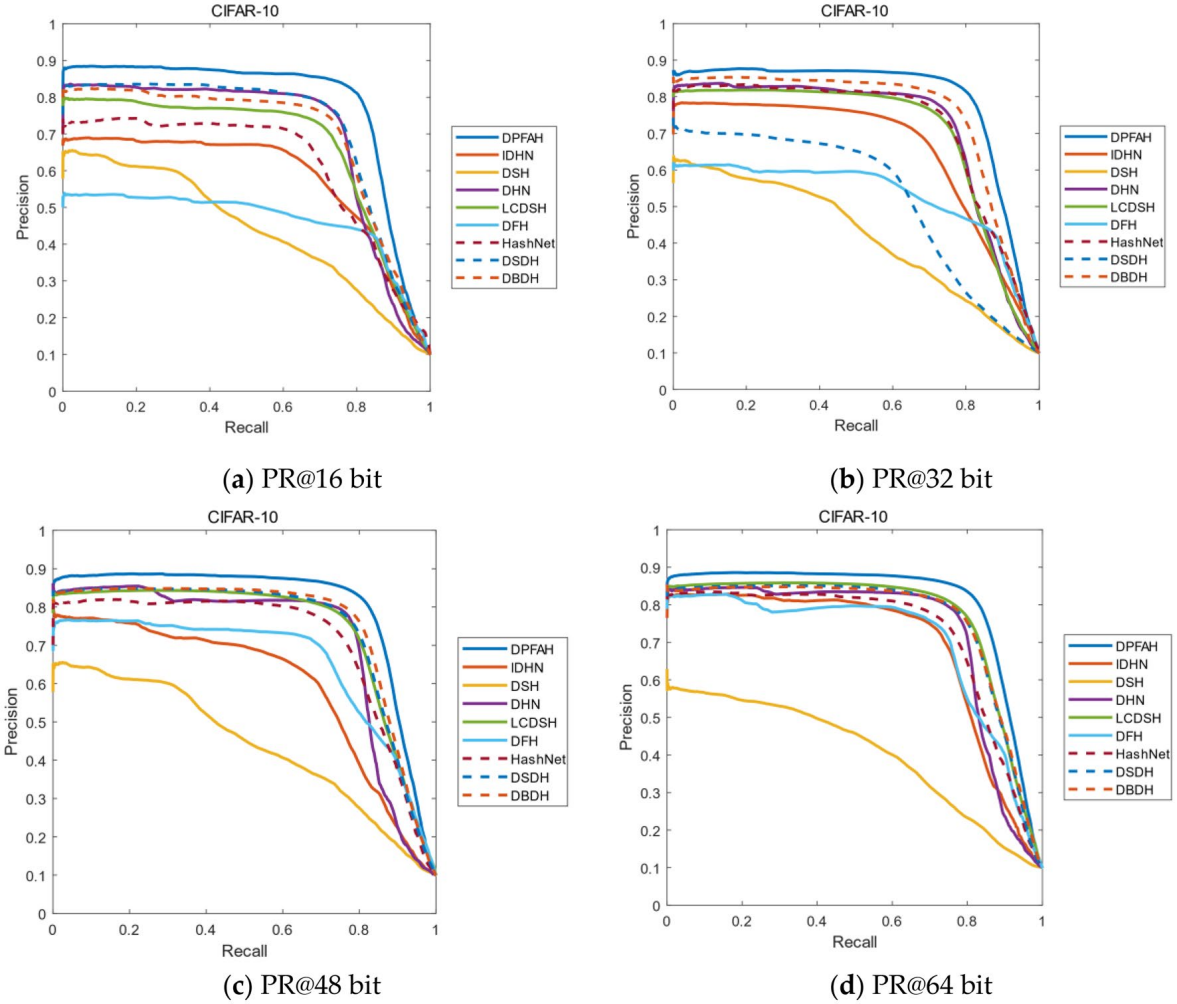
(**a–d**) The PR curves on CIFAR-10 of all methods with different bits. (Created by “matlab R2019a” url: https://ww2.mathworks.cn/products/matlab.html).

The curve of PR is an evaluation index with precision and recall as variables. Recall in the curve is set as the abscissa and precision is set as the ordinate. If the PR curve of one algorithm is completely surrounded by another algorithm, it can be asserted that the performance of the latter is better than that of the former. Therefore, the performance of the algorithm is judged by the area enclosed by the PR curve. Figure 8 shows the PR curves on dataset CIFAR-10. As can be seen from the Figure 8a–d, the curves of DPFAH method are significantly higher than all comparative methods. In particular, when the length of hash codes is 16bit, the enclosed area is much larger than DSDH, which has the best performance among all comparative methods.

**Figure 9:**
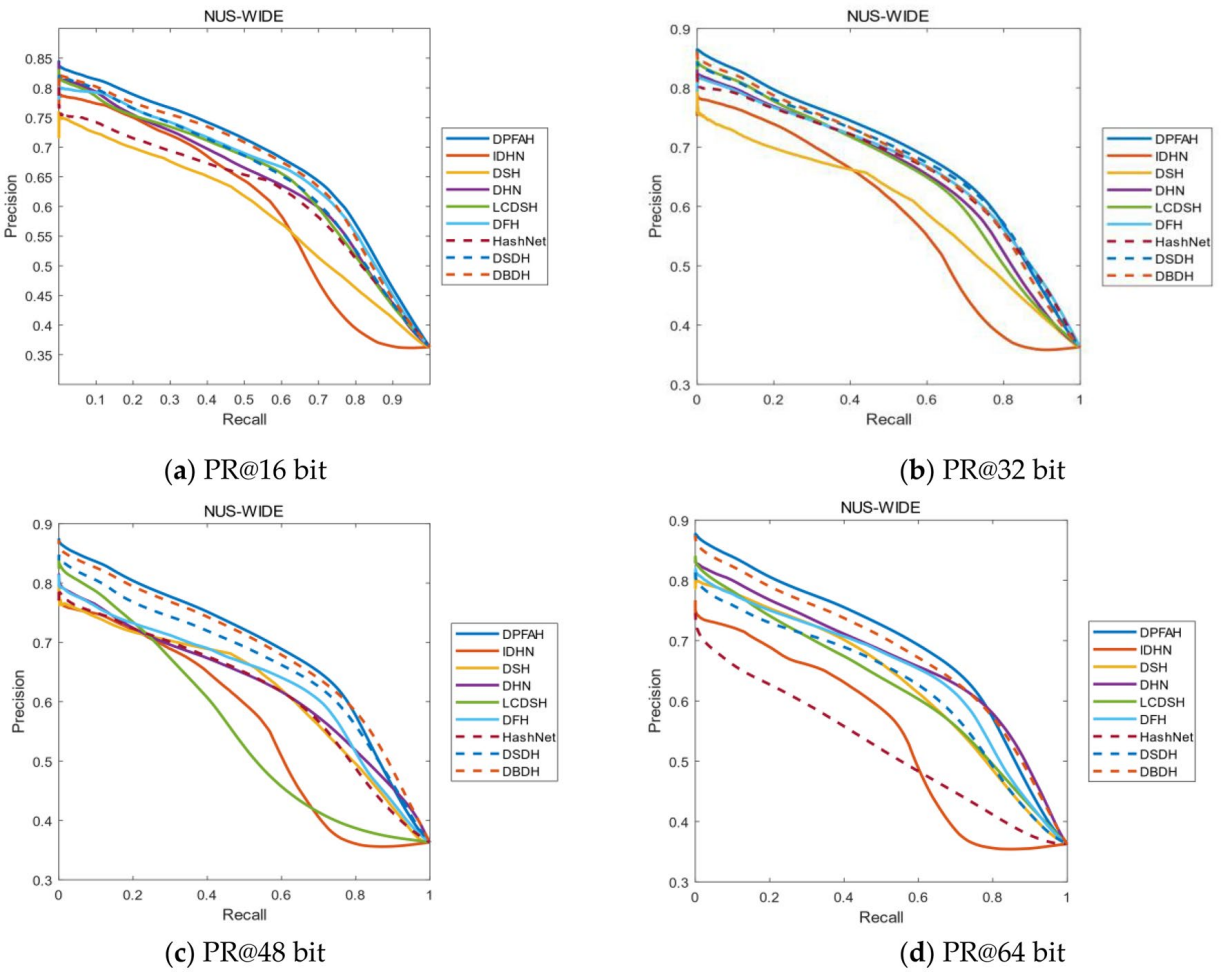
(**a–d**) Represent the PR curves on NUS-WIDE of all methods with different bits. (Created by “matlab R2019a” url: https://ww2.mathworks.cn/products/matlab.html).

Because NUS-WIDE is a multi-label dataset and the calculation process is relatively complex, the improvement on NUS-WIDE is not as obvious as that on CIFAR-10, but it is still the best of all methods. As shown in Figure 9, the mAP of DPFAH is the highest compared with the other eight comparison algorithms. In Figure 9a–d, DPFAH is higher than DSDH that has the best performance among all methods.

**Figure 10:**
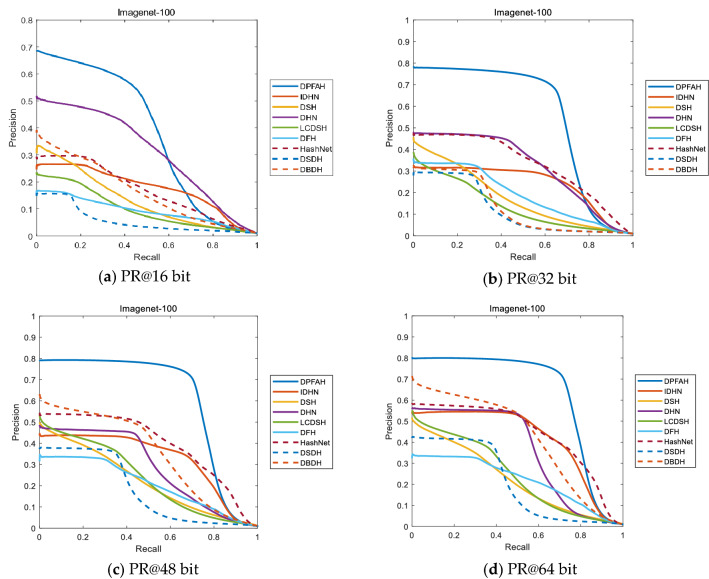
(**a–d**) Represent the PR curves on Imagenet-100 of all methods with different bits. (Created by “matlab R2019a” url: https://ww2.mathworks.cn/products/matlab.html).

Figure 10 shows the PR curves of 16, 32, 48 and 64 bits on Imagenet-100 dataset. The PR curve of DPFAH method is significantly higher than that of other comparison methods, especially on Fig. 10b–d In Fig. 10a, when recall is greater than about 0.6, the precision of DPFAH is less than that of DHN. When recall is less than 0.6, the precision of DPFAH is much higher than that of DHN. It can be seen from the overall PR curve siege area that DPFAH is significantly greater than DHN.

**Figure 11:**
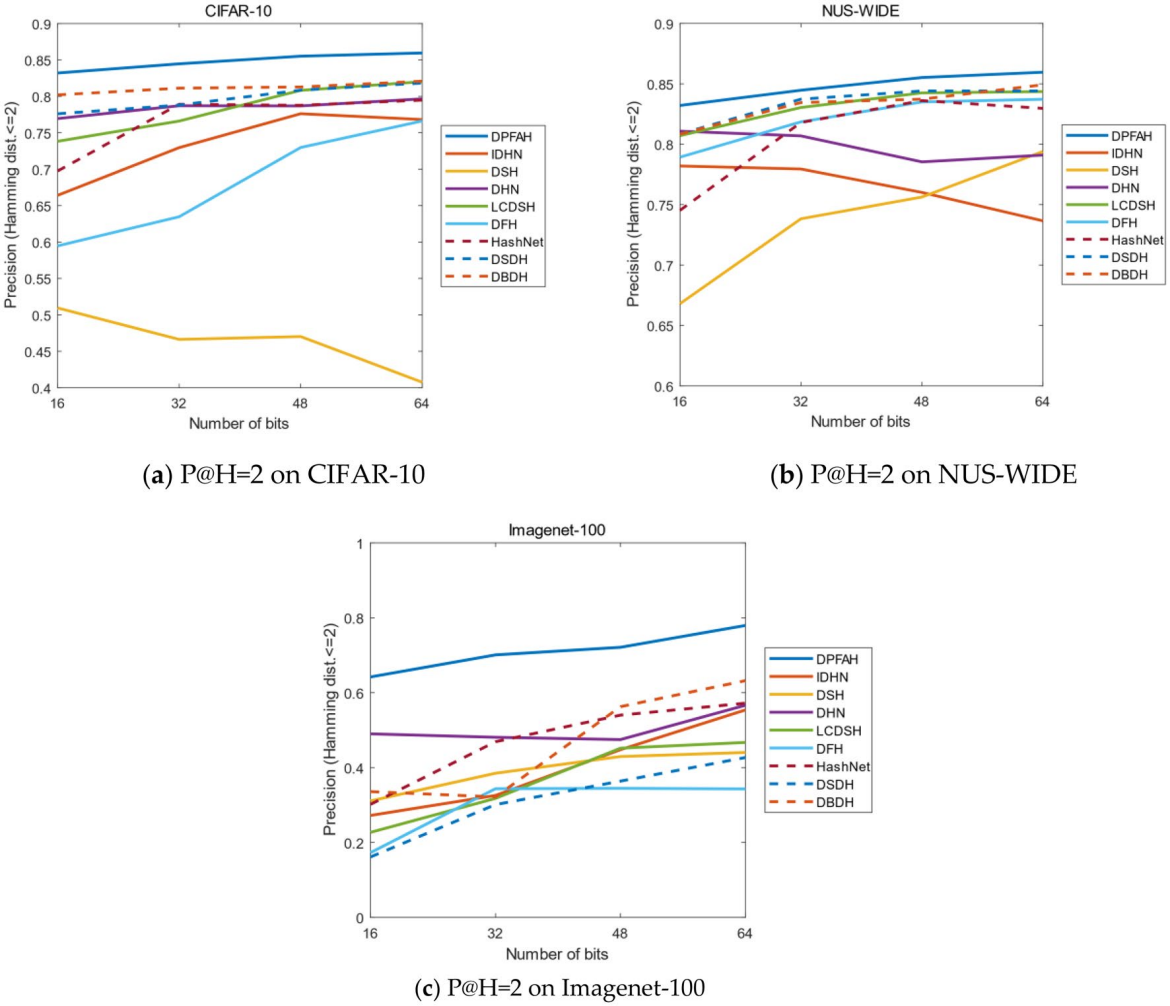
(**a–c**) Present P@H = 2 on three datasets. (Created by “matlab R2019a” url: https://ww2.mathworks.cn/products/matlab.html).

To achieve the aim that the hamming ranking only needs $\mathrm{\rm O}(1)$ time searches, the evaluating indicator P@H = 2 is important for the retrieval of hash codes. Figure 11 shows the result of P@H = 2 on three datasets, the method DPFAH obtains the highest precision in experiment. With the increase of hash code length, the precision also increases steadily, which shows that DPFAH model is more stable than the methods of DSH, IDHN and DHN on CIFAR-10, NUS-WIDE and Imagenet-100.

**Figure 12:**
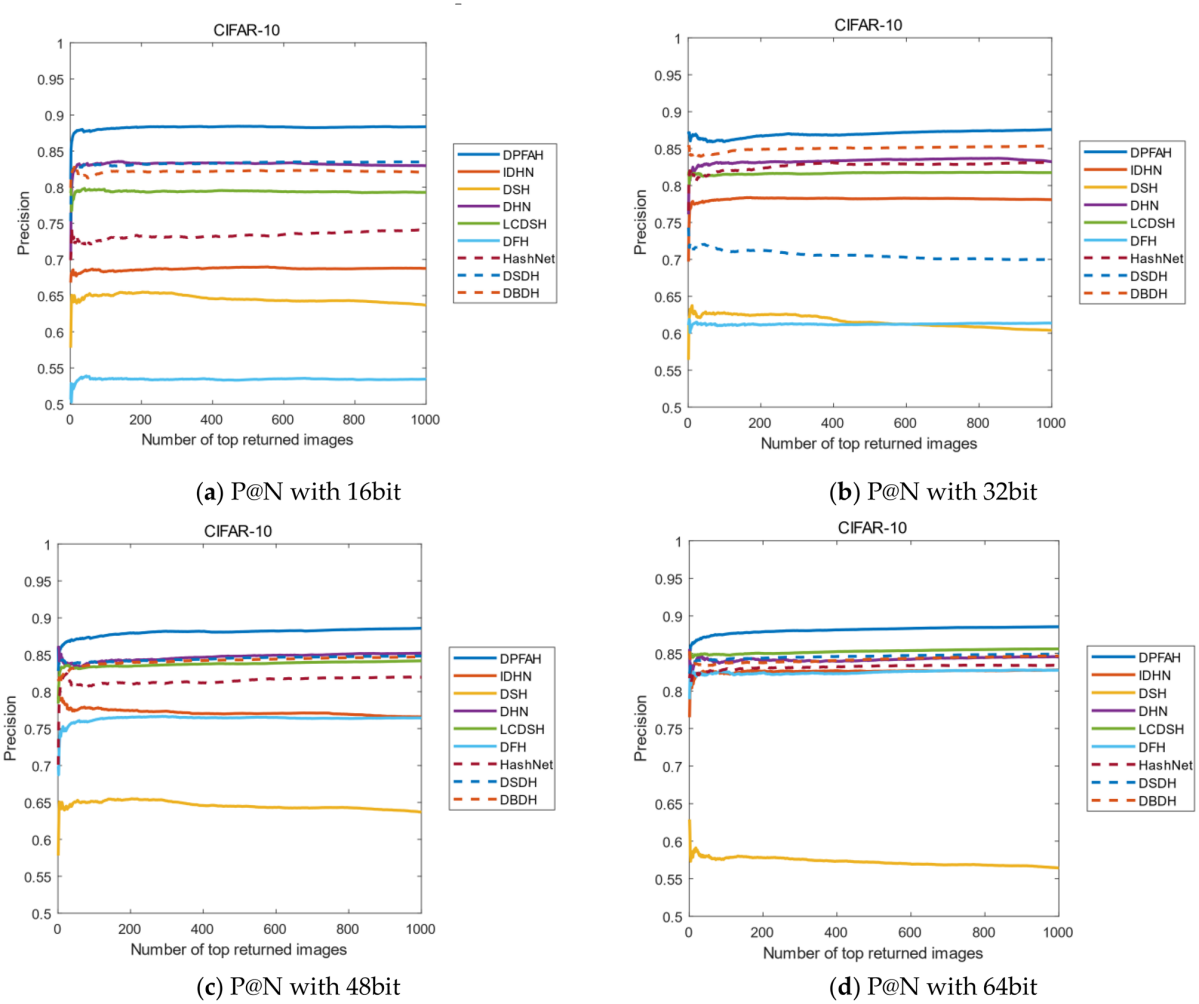
(**a–d**) Represent the P@N curves on CIFAR-10 of all methods with different bit. (Created by “matlab R2019a” url: https://ww2.mathworks.cn/products/matlab.html).

Another evaluation metric is the curves of P@N. The precision of the first 1000 images are selected in this experiment. Figure 12 shows the result of P@N on CIFAR-10 dataset, DPFAH method has achieved better precision than the other methods. Specifically, in Fig. 12a, the curves P@N of DPFAH is significantly higher than DHN and DSDH. In Fig. 12b–d, although the growth rate of DPFAH is not as obvious as Fig. 12a, the best precision is still obtained on 32bit, 48bit and 64bit.

**Figure 13:**
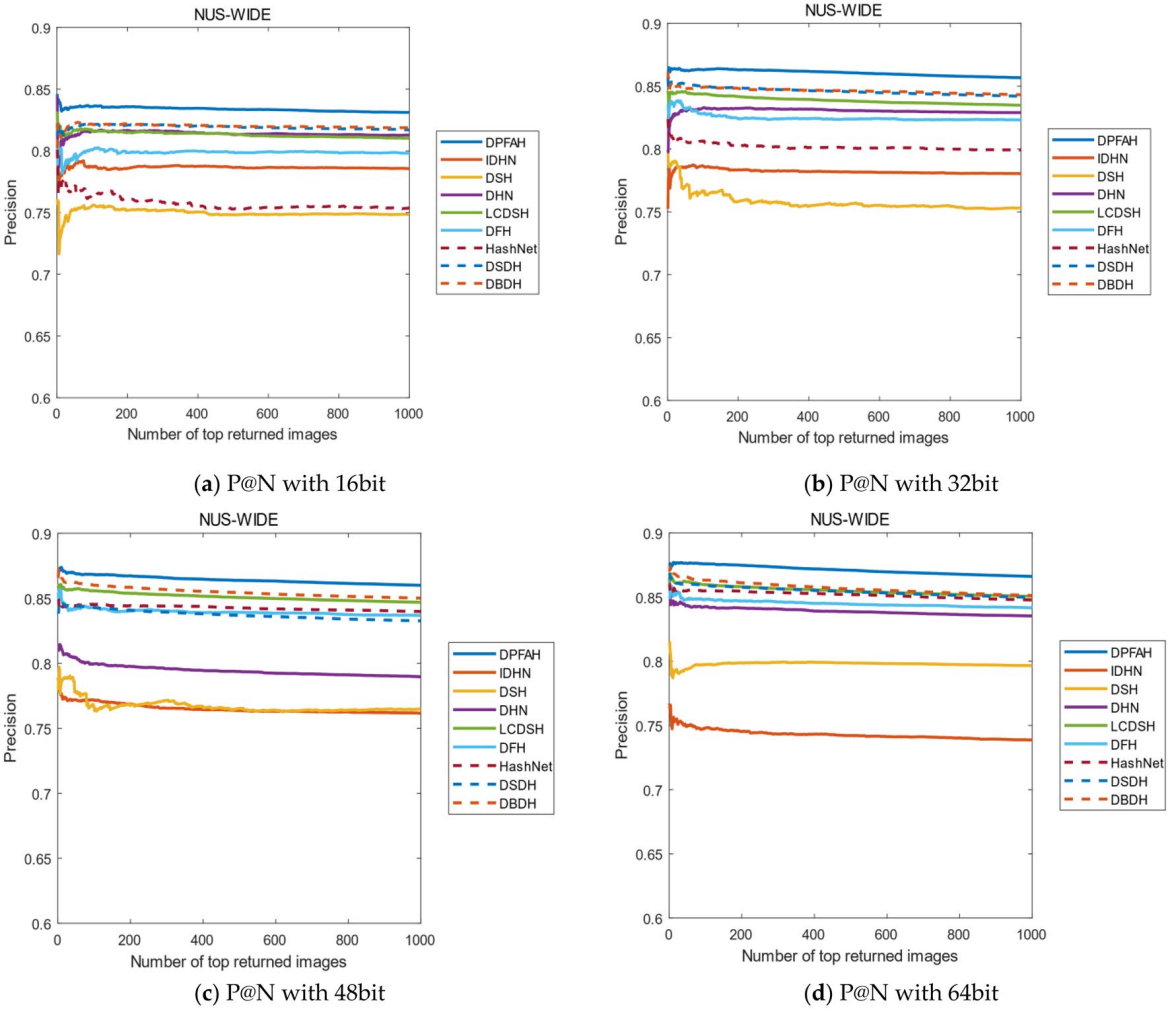
(**a–d**) Represent the P@N curves on NUS-WIDE of all methods with different bits. (Created by “matlab R2019a” url: https://ww2.mathworks.cn/products/matlab.html).

Figure 13 shows the P@N curves on NUS-WIDE, as can be from Fig. 13a,b, the P@N curves of all methods is relatively stable with the number of returned images increases. Compared with other algorithms, DPFAH still achieves the highest precision.

**Figure 14:**
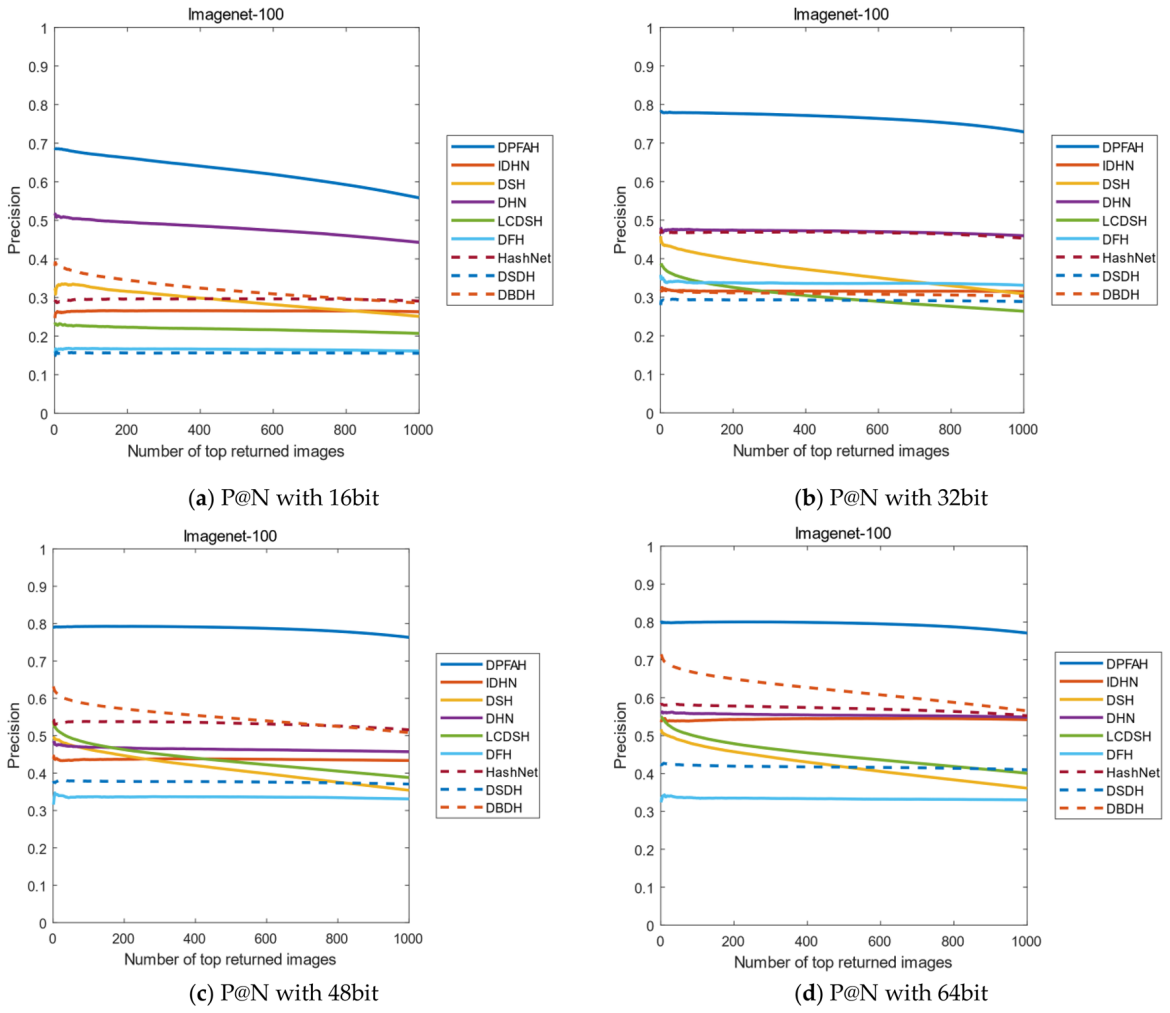
(**a–d**) Represent the P@N curves on Imagenet-100 of all methods with different bits. (Created by “matlab R2019a” url: https://ww2.mathworks.cn/products/matlab.html).

Figure 14 shows the P@N curves on the Imagenet-100 dataset. As can be seen from Figure 14b–d, when the length of the hash codes is 32, 48 and 64bits, the effect of DPFAH is obviously better than the other methods. With the increase of the number of images, the precision shows a stable trend, but the best results are still obtained in all comparison algorithms.

### Visualization show

**Figure 15:**
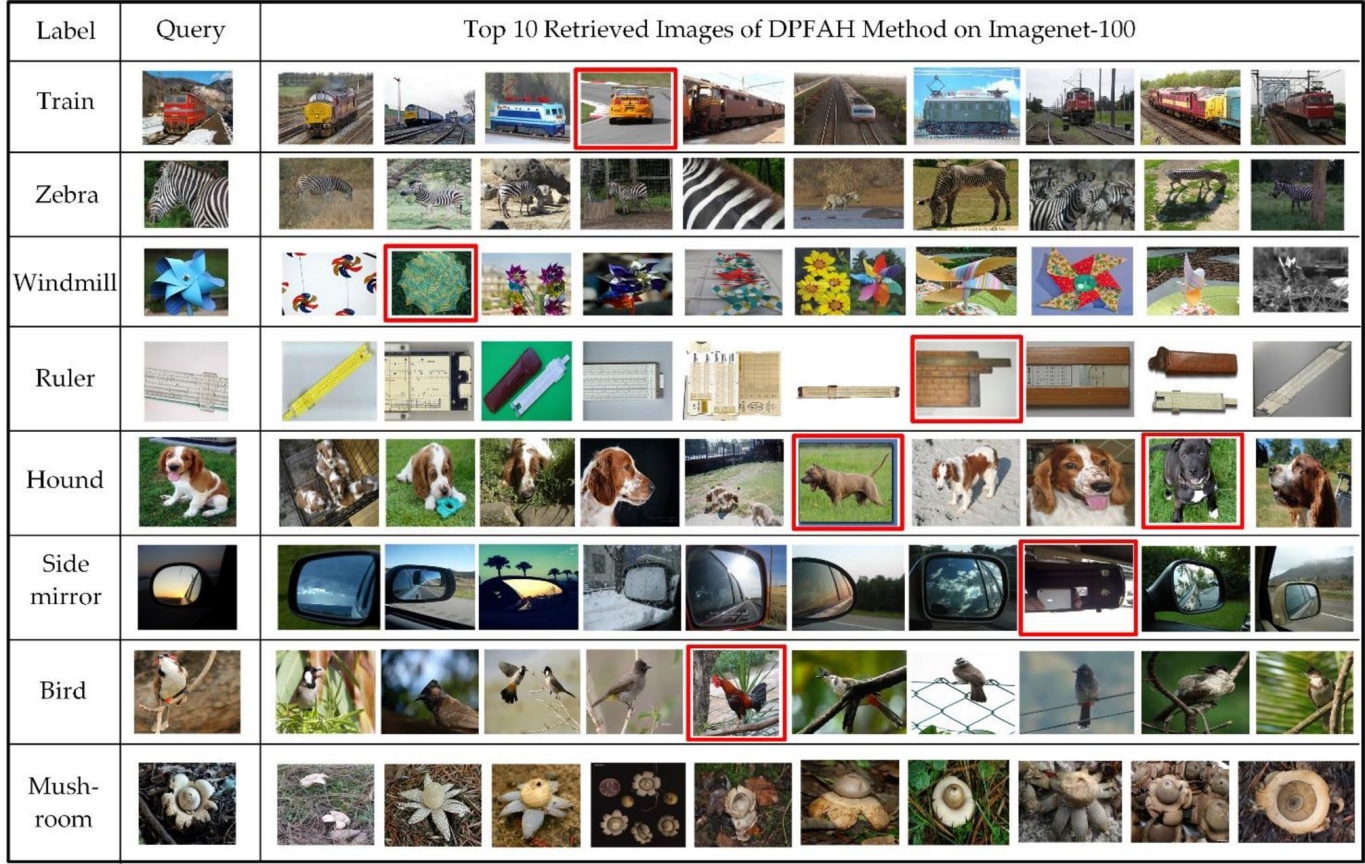
Top 10 retrieved results from Imagenet-100 dataset by DPFAH with 64bit hash codes. (Created by ‘Microsoft Office Visio 2013’ url: https://www.microsoft.com/zh-cn/microsoft-365/previous-versions/microsoft-visio-2013).

In Fig. 15, this paper visualizes the top 10 returned images of DPFAH for eight query images on Imagenet-100. The first row shows the label of the query images, the second row is query images, the retrieval results of DPFAH are shown at other rows. The red boxes are used to mark the false retrieval results.

## Conclusions

Existing image retrieval methods based on deep hashing have the defects of imbalance and insufficiency when existing hashing methods extract image features. Some scholars propose to employ channel-wise or spatial-wise attention mechanism into the network, which will add many parameters to the model and increase the computational complexity. Hence, this paper introduces a PFA module and propose DPFAH method. PFA module based on well-established suppression theory and define an energy function that determine the importance of each neuron. This module does not add any parameters to the network and directly extracts 3-D weight information of feature map. In addition, to generate accurate hash codes that retain the similarity information of the original image, this paper designs a classification branch to optimal network. The effectiveness of DPFAH method is proved by a large number of experiments. In particular, the evaluation index mAP increased by 2.95% when the PFA module is added in network. Hence, a better image retrieval model is obtained by DPFAH method.

## Data Availability

The CIFAR-10, NUS-WIDE and Imagenet-100 datasets are openly available at: http://www.cs.toronto.edu/kriz/cifar.html(accessed on 8 April 2022), http://lms.comp.nus.edu.sg/research/NUS-WIDE.html (accessed on 8 April 2022) and https://image-net.org (accessed on 8 April 2022).
